# The Effects of Isoflavone Supplementation Plus Combined Exercise on Lipid Levels, and Inflammatory and Oxidative Stress Markers in Postmenopausal Women

**DOI:** 10.3390/nu10040424

**Published:** 2018-03-29

**Authors:** Jéssica S. Giolo, Juliene G. Costa, Jair P. da Cunha-Junior, Ana Cláudia A. M. Pajuaba, Ernesto A. Taketomi, Adriele V. de Souza, Douglas C. Caixeta, Leonardo G. Peixoto, Erick P. de Oliveira, Sarah Everman, Foued S. Espindola, Guilherme M. Puga

**Affiliations:** 1 Laboratory of Cardiorespiratory and Metabolic Physiology, Federal University of Uberlândia, Uberlândia-MG 38400-678, Brazil; sanjuliaogiolo@hotmail.com (J.S.G.); julienegoncalves@hotmail.com (J.G.C.); 2 Laboratory of Immunotechnology and Immunochemistry, Institute of Biomedical Sciences, Federal University of Uberlândia, Uberlândia-MG 38400-902, Brazil; jair.cunha.junior@gmail.com (J.P.d.C.-J.); anapajuaba@gmail.com (A.C.A.M.P.); eat4y@yahoo.com.br (E.A.T.); 3 Laboratory of Biochemistry and Molecular Biology, Institute of Biotechnology, Federal University of Uberlândia, Uberlândia-MG 38400-902, Brazil; adriele_vds@hotmail.com (A.V.d.S.); caixetadoug@gmail.com (D.C.C.); lgpeixoto@yahoo.com.br (L.G.P.); foued@ufu.br (F.S.E.); 4 School of Medicine, Federal University of Uberlândia, Uberlândia-MG 38400-902, Brazil; erick_po@yahoo.com.br; 5 College of Graduate Health Studies, AT. Still University, Mesa, AZ 85206, USA; severman@atsu.edu

**Keywords:** aerobic and resistance exercises, soy protein, menopause, cytokines, anti-oxidant system

## Abstract

This study tested the effect of isoflavone supplementation in addition to combined exercise training on plasma lipid levels, inflammatory markers and oxidative stress in postmenopausal women. Thirty-two healthy and non-obese postmenopausal women without hormone therapy were randomly assigned to exercise + placebo (PLA; *n* = 15) or exercise + isoflavone supplementation (ISO; *n* = 17) groups. They performed 30 sessions of combined exercises (aerobic plus resistance) over ten weeks and consumed 100 mg of isoflavone supplementation or placebo. Blood samples were collected after an overnight fast to analyze the lipid profile, interleukin-6 (IL-6), interleukin-8 (IL-8), superoxide dismutase (SOD), total antioxidant capacity (FRAP), and thiobarbituric acid reactive substances (TBARS), before and after ten weeks of the intervention. There were no differences in the changes (pre vs. post) between groups for any of the inflammatory markers, oxidative stress markers or lipid profile variables. However, interleukin-8 was different between pre- and post-tests (*p* < 0.001) in both groups (Δ = 7.61 and 5.61 pg/mL) as were cholesterol levels (*p* < 0.05), with no interaction between groups. The combination of isoflavone supplementation and exercise training did not alter oxidative stress markers in postmenopausal women, but exercise training alone may increase IL-8 and decrease total cholesterol levels.

## 1. Introduction

The postmenopausal period is often associated with chronic diseases, which demonstrates the importance of prevention strategies. Low-grade chronic inflammation and oxidative stress are common changes in postmenopausal women [1,2], and regular physical exercise training and/or the consumption of phytoestrogens may be important strategies for minimizing or preventing these changes in women [3,4].

Resistance training combined with aerobic exercise has been shown to be effective for reducing reactive oxygen species and increasing antioxidant enzymes [5], as well as reducing total cholesterol levels in obese women more than aerobic exercise alone [6]. Regular exercise also plays an important role in increasing anti-inflammatory cytokines and reducing the release of pro-inflammatory agents by adipocytes by reducing visceral and total fat [4]. Interleukin-8 (IL-8) and interleukin-6 (IL-6) are pro-inflammatory cytokines, but they perform different and beneficial functions when released by skeletal muscle via the stimulus of physical exercise. IL-8 can induce angiogenesis and IL-6 can inhibit the release of pro-inflammatory tumor necrosis factor-alfa (TNF-α) and interleukin 1 beta (IL-1β), concurrently with an increase of anti-inflammatory cytokines IL-10 and IL-1ra, when released by exercise [7].

Among the phytoestrogens found in foods, soy isoflavones are frequently used as an alternative treatment for metabolic and cardiovascular diseases, and to ameliorate symptoms that appear during postmenopausal period in women, due to their similar structure and action to estradiol [8]. Some studies have shown that the consumption of 100 mg of isoflavones per day may reduce the reactive species, malondialdehyde, in postmenopausal women [3]. In addition, 138 mg of isoflavones along with 81 mg of dadzein increased the activity of the antioxidant enzyme, superoxide dismutase, in postmenopausal women [9]. Likewise, in animal models, these compounds have been shown to improve the lipid profile and total antioxidant capacity [10].

Due to the separate anti-inflammatory and antioxidant functions of physical exercise and isoflavones, our hypothesis was that the addition of isoflavone could induce greater and/or additive benefits to those found with exercise alone. A few studies have investigated the combined effect of isoflavone supplementation and exercise, but the results are still conflicting [11,12,13]. In fact, a clinical trial found improvements in risk factors, such as fat body mass, and inflammatory markers, such as C-reactive protein [11]. In addition, other studies showed reductions in low-density lipoprotein [13] and increased high-density lipoprotein with isoflavone supplementation and exercise in postmenopausal women [12]. In ovariectomized rats, this type of intervention was able to increase the total antioxidant capacity after 12 weeks [10]. However, few studies have investigated the effects of aerobic and resistance exercise in addition to isoflavone supplementation on inflammatory and oxidative stress markers in humans.

Therefore, the purpose of this study was to determine if the addition of isoflavone supplementation with combined aerobic and resistance exercise training has additional or better effects than exercise training alone on lipid profiles and inflammatory and oxidative stress markers in postmenopausal women.

## 2. Materials and Methods

This trial was a double-blind, randomized (electronic lottery), parallel prospective clinical trial conducted at the Laboratory of Cardiorespiratory and Metabolic Physiology at Physical Education Department at Federal University of Uberlândia, Uberlândia MG, Brazil. The study was approved by the local Ethics Committee for human studies (CAAE: 40622414.9.0000.5152), and all participants read and signed the informed consent before participation in the study. The experiments conformed to the principles set out in the World Medical Association Declaration of Helsinki. This research was registered at Clinicaltrials.gov (number: NCT03008785).

### 2.1. Participants

**Figure 1:**
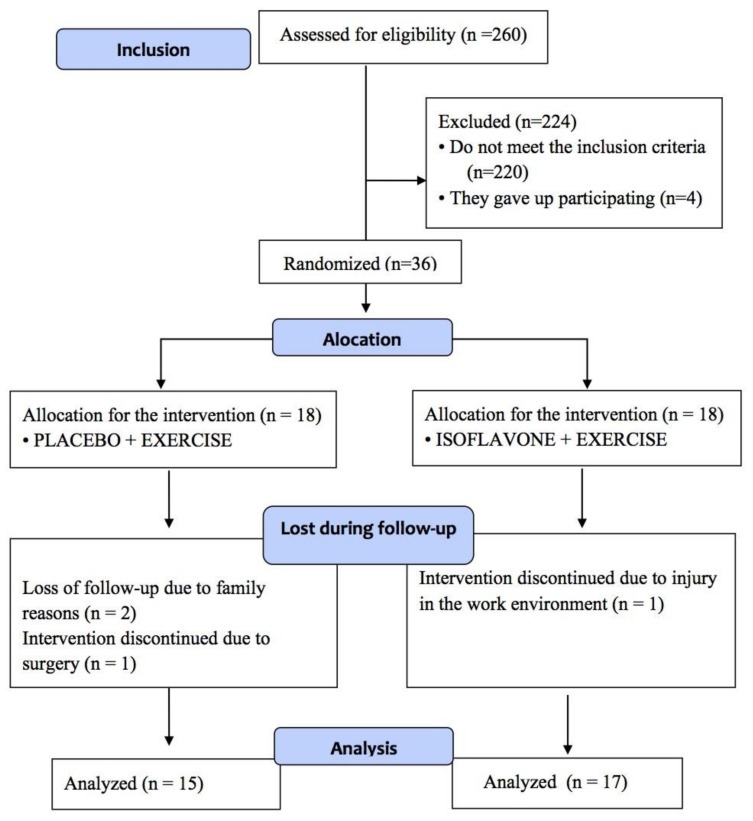
Flowchart with the distribution of the participants in the study.

This study included non-obese, postmenopausal women, aged between 50 and 70 years old. The inclusion criteria for the study were as follows: amenorrhea for at least 12 months; body mass index ≤30 kg/m^2^; ability to engage in treadmill and resistance exercises; no history of cardiovascular disease, diabetes or high blood pressure; no hormone therapy or soy derived supplementation; non smokers; and not using drugs that alter the lipid profile. Figure 1 presents the flowchart with the distribution of the participants in the study.

All participants who met the inclusion criteria were randomly assigned to two different groups: exercise training and placebo supplementation (PLA) or exercise training and isoflavone supplementation (ISO). The participants also underwent anthropometric measurements, fasting blood collection, an aerobic capacity and maximal strength test (1RM) and were familiarized with the exercises and ergometers used before testing. Blood samples were collected before and after 10 weeks of isoflavone or placebo supplementation and combined aerobic and resistance exercise training.

### 2.2. Supplementation

The participants in the ISO group consumed one capsule per day containing 100 mg of isoflavones (Xi’An Green-Life Natural Products, Xian, Shaanxi, China) 33 mg of genistein, 93.5 mg of dadzein and 3.2 mg of glycitein derived from soybean and corresponding approximately 37.58 g of soy [14]. The plasma half-life of isoflavone is approximately 6–8 h [15,16], however metabolites derived after its ingestion may be elevated in the urine for 24 h and return to baseline in the next 48–72 h [17]. The participants in the PLA group received 100 mg of cornstarch daily in a single capsule. All capsules were identical in appearance.

### 2.3. Body Composition and Anthropometric Measures

Body mass was measured using a Micheletti (São Paulo, SP, Brazil) electronic scale and height was measured using a standard stadiometer Sanny (São Paulo, SP, Brazil), followed by body mass index (BMI) calculation. Total fat mass and fat-free mass were measured by bioimpedance (Biodynamics model 450c—Biodynamics, Shoreline, WA, USA). This analysis was performed in the morning after participants had fasted for at least 8 h, and hydration was controlled as previously reported by de França et al. [18].

### 2.4. Dietary Assessment

All participants were instructed to maintain their regular diet during the study. Food intake was evaluated at baseline and at the end of the study by three dietary recalls collected by trained nutritionists. Dietary data were reported on non-consecutive days (two week days and one weekend day), and were analyzed by Dietpro^®^ 5.7i (Minas Gerais, MG, Brazil) software and by the United States Department of Agriculture (USDA) food composition table.

### 2.5. Exercise Training

The exercise training consisted of combined aerobic and resistance exercises performed during the same session. All women performed 30 sessions over ten weeks on non-consecutive days three times per week, and all sessions lasted 50 min, including 5 min of warm-up on a treadmill, 20 min of aerobic exercise on a treadmill, 20 min of resistance exercises and 5 min of cool down exercises. The order of aerobic and resistance exercises was changed every training session. Before the exercise training intervention, all women performed a familiarization session with the treadmill and resistance exercises.

Aerobic training was performed on treadmill at a speed of 5.5 km/h, and exercise intensity was imposed only by increasing the treadmill incline. The training intensity was between ventilatory threshold 1 (VT1) and ventilatory threshold 2 (VT2), determined previously by an incremental test. The incremental test was adapted from Puga et al. [19]. Briefly, women performed a sub-maximal incremental test on treadmill at a fixed speed of 5.5 km/h and the intensity was added by increasing the treadmill incline (%). The test started with a 1% incline and was increased in increments of 1% every 2 min until the volunteers reached 85% of their predicted maximum heart rate and/or a perceived rate of exertion of 18 on the Borg Scale. The VT1 and 2 were identified based on the ventilatory equivalents for oxygen (VE/VO_2_ ratio) and carbon dioxide (VE/VCO_2_ ratio) responses using a gas analyzer (Cosmed Quark CPET, Rome, Italy) in accordance with Wasserman [20].

The resistance training was performed as 2 sets of 15 repetitions, with an interval of 30 s between exercises. The exercises consisted of seven exercises for large muscle groups, including 45° leg press, crossover seated row, bench press, pec deck, lateral pull-down, squat with fitball and abdominal exercises. The intensity of the exercises was 60%, based on the 1RM test, with load readjustments via a new test in the 5th week. The 1RM test started with a warm up that consisted of two sets of exercises at 40–50% (5–10 repetitions) and 60–80% (3–5 repetitions) of the participant’s estimated 1RM. The test began with volunteers performing one complete repetition (eccentric and concentric phase) at maximal effort. The 1RM consisted of the workload being performed with no more than one repetition [21].

### 2.6. Blood Samples Collection and Analysis

Fifteen mL blood samples were collected after an overnight fast, five days before, and 72 h after, the last exercise training session to eliminate the acute effects of the exercise. These samples were placed in EDTA or serum tubes with separator gel and then centrifuged at 3000 rpm for 15 min and stored in small tubes for future analysis.

Plasma concentrations of total cholesterol, triglycerides, high density lipoprotein (HDL) and low density lipoprotein (LDL) cholesterol, uric acid and glucose were determined by enzymatic colorimetric methods. The concentration of glycated hemoglobin (HbA1c) was determined by the turbidimetry method. All analyses were performed using an automated system (Cobas Mira, Roche Instruments Inc. Bellport, NY, USA), using commercial kits (Labtest, Minas Gerais, Brazil). Very low density lipoprotein (VLDL) values were calculated using the Friedwald equation.

Human cytokines (IL-8, IL-1β, IL-6, TNF, IL-10 and IL-12p70) in serum samples were measured by the flow cytometry technique (FACSCantoII BD™, San Jose, CA, USA) using a Human Inflammatory Cytokines Kit (BD™ Cytometric Bead Array (CBA), BD Biosciences, San Jose, CA, USA) according to the manufacturer’s instructions.

The total antioxidant capacity in the plasma was evaluated by the Ferric-Ability of Plasma (FRAP) methodology and calculated from the standard trolox curve, as described by Justino et al. [22]. The activity of the superoxide dismutase enzyme (SOD) was determined based on the auto-oxidation capacity of pyrogallol. Lipid peroxidation levels were analyzed by the TBARS (thiobarbituric acid reactive substances) method, using a curve of 1,1,3,3-tetramethoxypropane (TMP) as the standard [22].

### 2.7. Statistics

The results are presented as means ± standard errors. The data distribution was analyzed using the Shapiro-Wilk test. Sample size was calculated using data from GPower 3.0.10, using an α value of 0.05, a β value of 0.15 and a power analysis of 85% with a final number of volunteers of 30 women. The two-way Analysis of Variance (ANOVA) for repeated measures was used to analyze the time (pre and post) and group (PLA and ISO) interactions with a Bonferroni post hoc test, when appropriate. Unpaired student’s *t*-tests were used to compare the delta values between groups. A Pearson correlation was also performed. A *p*-value of ≤0.05 was used for statistical significance, and all statistical analyses were performed using SPSS software version 20.0 (IBM, NewYork, NY, USA).

## 3. Results

The included women had mean ages of 52.7 ± 4.9 and 56.0 ± 5.4 years; body masses of 64.7 ± 2.2 and 65.4 ± 2.1 kg; body mass indices of 26.9 ± 0.7 and 26.4 ± 0.8 kg/m^2^ and fat masses of 35.4 ± 1.1 and 35.4 ± 1.1% in the PLA and ISO groups, respectively. There were no significant differences in any of these characteristics between groups at the beginning of the study. There were also no significant differences between pre-values (described above) and post values in body mass (65.6 ± 2.3 kg for PLA and 64.3 ± 2.1 kg for ISO), body mass index (26.9 ± 0.7 8 kg/m^2^ for PLA and 26.4 ± 0.8 8 kg/m^2^ for ISO), and fat mass (34.6 ± 1.2 kg for PLA and 35.5 ± 1.3 kg for ISO).

The volunteers exercised at an average intensity of 5 ± 5% (treadmill inclination), a rate of perceived exertion of 16 ± 2 and a heart rate zone of 158 ± 16 bpm. There were no significant differences in dietary protein, fat or carbohydrate ingestion evaluated by food recall during the 10-week intervention between the groups.

Vitamins A, C and D, which are considered exogenous non-enzymatic antioxidants, were also evaluated by food recall and were not different between groups or moments during the intervention. The consumption of vitamin A in the PLA group was 224.82 ± 368.28 mg pre-intervention and 217.82 ± 376.82 mg post-intervention, and in the ISO group, 147.57 ± 128.53 mg pre-intervention and 113.08 ± 60.05 mg post-intervention. Vitamin C consumption in PLA was 224.82 ± 368.28 mg pre-intervention and 217.82 ± 376.82 post-intervention, and in the ISO group, 147.57 ± 128.53 pre-intervention and 113.08 ± 60.05 post-intervention. The intake of vitamin D in the PLA group was 46.21 ± 33.11 mg pre-intervention and 44.69 ± 31.36 mg post-intervention, and in the ISO group, 49.91 ± 42.76 mg pre-intervention and 51.48 ± 40.16 mg post-intervention. There were also no differences between moments or groups in carbohydrate (pre: 221.02 ± 45.87 g and post: 204.65 ± 63.85 g for PLA group and pre: 203.98 ± 42.21 and post: 193.17 ± 48.98 g for ISO group), protein (pre: 67.25 ± 16.83 g and post: 60.84 ± 20.44 g for PLA group and pre: 61.61 ± 16.40 g and post: 56.97 ± 15.63 g for ISO group) lipid (pre: 80.14 ± 26.34 g and post: 68.05 ± 17.85 for PLA group and pre: 64.90 ± 17.13 g and post: 61.42 ± 18.83 for ISO group) or fiber intake (pre: 15.97 ± 6.24 g and post: 12.79 ± 4.94 g for PLA group and pre: 15.49 ± 6.04 g and post: 13.50 ± 4.94 g for ISO group).

The fasting lipid profile, glucose, glycated hemoglobin (HbA1C) and uric acid levels were analyzed before and after the 10-week intervention in both groups and are shown in Table 1. No significant differences were found for any of the variables for time or group. Although total cholesterol levels increased after the intervention in both groups with a significant effect of time (*p* = 0.04), no interaction between the groups was found (group * moment) (*p* = 0.07). The delta values (post–pre values) were also not different between groups.

Table 2 shows the levels of inflammatory and oxidative stresses markers analyzed before and after the 10-week intervention in both groups. Among the possible cytokines to be analyzed in the Kit (IL-8, IL-1β, IL-6, TNF, IL-10 and Il-12p70), only interleukin-6 (IL-6) and interleukin-8 (IL-8) were read-sensitive. We found no differences between time or group in any of the inflammatory markers or oxidative stress. However, IL-8 concentrations increased after the intervention in both groups with a significant effect of time (*p* = 0.001), but no interaction between the groups was found (group * moment) (*p* = 0.55). The deltas of the variables (post–pre values) were not different between groups.

We found a significant positive Pearson correlation between the TBARS and FRAP variables (*r* = 1.0, *p* < 0.0001) in both groups after the intervention. A significant negative correlation between IL-8 and FRAP (*r* = 0.76, *p* = 0.0009) and between IL-8 and TBARS (r = 0.76; *p* = 0.0009) was observed only in the PLA group after the intervention. No other significant correlations were found among all the other variables analyzed. No changes were found to be significant between the groups.

## 4. Discussion

The present study examined the effect of isoflavone supplementation in addition to a combined aerobic and resistance training program on lipid levels, inflammatory markers and oxidative stress in postmenopausal women. Our results indicate that the supplementation of isoflavones when combined with exercise training does not promote additive changes in any of the inflammatory markers or oxidative stress markers measured in postmenopausal women. However, combined training was effective in reducing total cholesterol and increasing IL-8 levels in non-obese, postmenopausal women.

The increase in IL-6 concentration caused by acute exercise (after one exercise session) is believed to inhibit the release of pro-inflammatory agents, such as TNF-α, and stimulate immune cells to produce anti-inflammatory cytokines by promoting a chronic anti-inflammatory effect, when exercise is practiced regularly [7]. In our study, we found no significant difference in serum IL-6 concentration after the intervention in either group. Lebon et al. [11] that also found no change in IL-6 concentration after 6 months of supplementation of 70 mg of isoflavones and combined exercise in healthy postmenopausal women. Likewise, Ho et al. [23] did not observe a change in IL-6 after performance of any of the types of exercises studied, but TNF-α levels decreased more sharply after 12 weeks in a group that performed combined moderate intensity exercise compared to groups that performed isolated aerobic and resistance exercises. On the other hand, the study conducted by Forti et al. [24] found reductions in IL-6 concentrations in healthy young participants. This study showed that nine weeks of resistance training increased IL-8 and decreased IL-6, which demonstrated that this kind of training can have anti-inflammatory effects in healthy, young people.

Balducci et al. [25] showed that, in people with metabolic syndrome and type 2 diabetes, high-intensity training, performed over a 12-month period, reduced IL-6 concentrations in patients who performed combined aerobic and resistance exercises. These results are not in agreement with our results, and we believe that those who already have elevated levels of inflammatory markers are more likely to see improvements after an exercise training program. Although postmenopausal women may present higher concentrations of IL-6 compared to premenopausal women [26], our volunteers were healthy and had low baseline serum levels of IL-6, which may explain the absence of changes after 10 weeks of study.

IL-8, when released by skeletal muscle through exercise, acts as an important angiogenic factor which is separate from its pro-inflammatory capacity [7]. In a recent study, Forti et al. [24] analyzed different intensities of resistance exercise training in healthy young participants for 9 weeks and found an increase in IL-8 concentrations in participants in a high-intensity exercise group and in low-resistance training with maximal effort group, but not in a low intensity with low effort exercise group. This response was associated with adaptation to intense exercise and may be responsible for the induction of angiogenesis. Alternatively, Izzicupo et al. [27] found a reduction in serum IL-8 levels after a 13-week moderate intensity walking program in postmenopausal women. Although the authors found a reduction in IL-8 concentrations rather than an increase, the angiogenic properties found in the serum improved after the study [27]. In our study, we observed a significant increase in serum IL-8 in both groups after the 10-week intervention, but there was no difference between groups. Independent of isoflavone supplementation, combined exercise training, with the complementary effects of both exercise components (aerobic and resistance) at a moderate intensity, increases IL-8 levels and this may be related to the induction of angiogenesis, since skeletal muscle, with stimulation from exercise, may induce the production of IL-8 and increase the expression of its receptor responsible for angiogenesis [7].

The period after menopause also contributes to an imbalance in the pro and antioxidant systems [2]. In fact, acute exercise can increase reactive oxygen species production, but chronic exercise training contributes to an improvement in antioxidant defenses and consequently, reduces the state of oxidative stress [28]. One study, using a combined exercise training intervention over 12 weeks in women with fibromyalgia, found a reduction in plasma TBARS and an increase in serum levels of the antioxidant enzyme, catalase [5]. Similarly, the consumption of 100 mg of isoflavones (with no exercise intervention) per day may reduce the concentration of the reactive species, malondialdehyde [3], the intercellular adhesion molecule (ICAM-1) and E-selectin, in postmenopausal women [29]. So, to our knowledge, this is the first study to investigate the association of isoflavones with a combined exercise program for oxidative stress markers in postmenopausal women, and our results indicate that 100 mg of isoflavones in addition to moderate combined exercise does not alter these markers after 10 weeks in this population. We believe that a longer time frame may be necessary to detect the effects of this type of intervention on oxidative stress variables in healthy postmenopausal women.

Maintaining a healthy lipid profile is very important, since elevated levels of total cholesterol are directly related to cardiovascular disease [30]. Mann et al. [31] reviewed the effects of different types of exercise training on cholesterol levels and concluded that resistance training may complement the effects of aerobic training, although there is limited literature comparing the three types of exercises (aerobic, resistance and combined exercises). Likewise, Park et al. [6], showed that combined exercise may reduce total cholesterol in obese women more than in aerobic exercise alone. Therefore, the reduction of total cholesterol found in both groups in our study is in agreement with previous clinical trials and indicates that combined physical exercise may be an alternative for reducing this risk factor in postmenopausal women.

On the other hand, the effect of isoflavone consumption on cholesterol levels is still controversial. According to a meta-analysis by Zhan and Ho [32], isoflavone supplementation is associated with improvements in the lipid profile, although other authors have not found the same isoflavone effect on lipid parameters [33,34]. When isoflavones are taken in combination with exercise training, the effect on the lipid profile remains inconclusive. Some authors did not observe the additive effects of these compounds and exercise training on lipid variables [35,36]. However, Wu et al. [12] found an increase in HDL after 24 weeks of 75 mg of isoflavones combined with aerobic exercise and concluded that some benefits of this association can be depend on the production of equol, a specific metabolite of daidzein produced by bacteria in the gut. This metabolite is not found in soya, but is formed by intestinal flora in 30–50% of individuals after consumption of these compounds [37]. In the present study, we found no interaction between group or time for total cholesterol, and this suggests that isoflavone supplementation had no additive beneficial effects on exercise-mediated responses. The mechanisms behind a possible lack of interaction between exercise and isoflavone consumption are still unclear.

There were some limitations in the present study. The study was conducted in generally healthy, non-obese women; therefore, the results might not be applicable to other groups receiving treatment with higher potency medication or for longer than 10 weeks. It is important to note also that this result is applicable only for isoflavone supplementation and may not be extrapolated to isoflavone consumption from natural and regular food. Other limitations were the absence of an evaluation of isoflavone levels in the urine to detect how much soy protein was actually absorbed, as well as the 10-week intervention, which is a biologically acceptable time frame for a clinical study but may have been relatively short for detecting the effects of isoflavone supplementation associated with combined exercise. More studies are needed with larger numbers of participants and with patients with dyslipidemia, obesity or with high oxidative stress levels or inflammatory states to investigate the effects of these interventions.

## 5. Conclusions

Isoflavone supplementation did not promote additive or independent effects compared to the combination of aerobic and resistance exercises alone on the lipid profile and on inflammatory and oxidative stress markers in non-obese postmenopausal women. However, this exercise training program increased IL-8 levels independent of the isoflavone supplementation and appears to be effective for reducing total cholesterol in this population.

## Figures and Tables

**Table 1:** Comparison of lipid profile, glucose, glycated hemoglobin and uric acid between the pre- and post-interventionin the placebo (PLA) and isoflavone (ISO) groups.

	Pre	Post	Δ	*p*	*p*	*p*
	Mean ± SE	Mean ± SE		(Groups)	(Moments)	(Groups * Moments)
Total Cholesterol (mg/dL)						
PLA	210 ± 8	197 ± 7	−13.07 ± 3.72	0.34	0.04	0.07
ISO	215 ± 9	214 ± 9	−1.13 ± 5.03			
LDL (mg/dL)						
PLA	126 ± 9	118 ± 6	−7.54 ± 4.29	0.34	0.24	0.21
ISO	133 ± 8	133 ± 8	0.25 ± 4.21			
HDL (mg/dL)						
PLA	55.4 ± 3.6	50.5 ± 3.0	−4.54 ± 1.27	0.25	0.07	0.16
ISO	58.1 ± 3.4	57.5 ± 2.6	−0.59 ± 2.16			
VLDL (mg/dL)						
PLA	24.7 ± 3.1	23.5 ± 3.0	−1.20 ± 1.60	0.77	0.91	0.47
ISO	22.7 ± 2.1	23.6 ± 2.0	0.88 ± 2.17			
Triglycerides (mg/dL)						
PLA	124 ± 16	118 ± 15	−6.00 ± 7.98	0.77	0.91	0.47
ISO	113 ± 11	118 ± 10	4.38 ± 10.86			
Glucose (mg/dL)						
PLA	86.6 ± 1.7	86.8 ± 1.6	0.17 ± 1.33	0.92	0.27	0.20
ISO	87.5 ± 1.0	85.5 ± 1.4	−2.08 ± 1.09			
Glycated Hemoglobin (%)						
PLA	5.50 ± 0.07	5.52 ± 0.10	0.02 ± 0.06	0.24	0.53	0.92
ISO	5.60 ± 0.05	5.63 ± 0.03	0.03 ± 0.05			
Uric Acid (mg/dL)						
PLA	4.5 ± 0.3	4.6 ± 0.3	0.04 ± 0.13	0.79	0.68	0.40
ISO	4.5 ± 0.2	4.4 ± 0.1	−0.12 ± 0.14			

PLA: placebo and exercise group; ISO: isoflavones and exercise group; LDL: Low density lipoprotein; HDL: High density lipoprotein; VLDL: Very low density lipoprotein.

**Table 2:** Comparison of inflammatory and oxidative stress markers in the pre and post intervention moments in the placebo (PLA) and isoflavone (ISO) groups.

	Pre	Post	Δ	*p*	*p*	*p*
	Mean ± SE	Mean ± SE		(Groups)	(Moments)	(Groups * Moments)
Interleukin-8 (pg/mL)						
PLA	13.70 ± 1.91	21.31 ± 2.23	7.61 ± 2.50	0.92	0.001	0.55
ISO	14.52 ± 1.10	20.13 ± 1.79	5.61 ± 2.23			
Interleukin-6 (pg/mL)						
PLA	1.54 ± 0.39	1.30 ± 0.32	−0.24 ± 0.27	0.76	0.22	0.98
ISO	1.42 ± 0.21	1.19 ± 0.26	−0.23 ± 0.25			
FRAP (umol/L eq. Trolox)						
PLA	124.31 ± 5.24	123.18 ± 5.67	−1.13 ± 3.48	0.65	0.39	0.73
ISO	122.47 ± 2.83	119.85 ± 3.03	-2.63 ± 2.58			
TBARS (umol/L TBA-RS)						
PLA	24.65 ± 1.08	24.42 ± 1.17	−0.23 ± 0.72	0.72	0.38	0.70
ISO	24.40 ± 0.61	23.82 ± 0.66	−0.58 ± 0.56			
SOD (SOD/mg prot)						
PLA	0.190 ± 0.007	0.194 ± 0.008	0.003 ± 0.010	0.20	0.24	0.50
ISO	0.176 ± 0.007	0.193 ± 0.008	0.012 ± 0.008			

PLA: placebo and exercise group; ISO: isoflavones and exercise group; FRAP: plasma ferric reduction capacity; TBARS: thiobarbituric acid reactive substances; SOD: superoxide dismutase.
